# Enhancement of Emodin Production by Medium Optimization and KH_2_PO_4_ Supplementation in Submerged Fermentation of Marine-Derived *Aspergillus favipes* HN4-13

**DOI:** 10.3390/md19080421

**Published:** 2021-07-26

**Authors:** Xiaohan Qiu, Lizhi Gong, Xiujuan Xin, Faliang An

**Affiliations:** Key Laboratory of Bioreactor Engineering, East China University of Science and Technology, 130 Meilong Road, Shanghai 200237, China; y30180978@mail.ecust.edu.cn (X.Q.); 15216603669@163.com (L.G.); xinxj@ecust.edu.cn (X.X.)

**Keywords:** emodin, *Aspergillus favipes* HN4-13, KH_2_PO_4_, submerged fermentation

## Abstract

Emodin is a widely distributed anthraquinone derivative with a variety of biological activities, one that can be efficiently produced by marine-derived fungus *Aspergillus favipes* HN4-13. However, its relatively low fermentation yield limits further development and pharmaceutical research work. In this study, Plaekett–Burman design and central composite design were adopted to optimize the fermentation conditions of *A*. *favipes* HN4-13. Optimal fermentation conditions in a 250-mL Erlenmeyer flask with 50 mL of medium were 59.3 g/L soluble starch, 10 g/L yeast extract paste, 30 g/L seawater salt, 1.04 g/L KH_2_PO_4_, 0.05 g/L MgSO_4_·7H_2_O, 0.01 g/L FeSO_4_·7H_2_O, seed culture 24 h, pH 5, inoculum size 18%, culture temperature 32 °C, and shaking at 160 rpm/min for 7 days. The production of emodin could achieve 132.40 ± 3.09 mg/L, with no significant difference from the predicted value (132.47 mg/L). Furthermore, KH_2_PO_4_ supplementation strategy was employed to regulate the mycelial morphology, upregulate the transcriptional level of biosynthesis gene cluster, and enhance emodin production (185.56 ± 4.39 mg/L).

## 1. Introduction

Emodin is one of the most widely distributed anthraquinone derivatives, being famous for its medicinal value and various biological activities including anti-bacterial [1] and anti-inflammatory [2] effects, cell proliferation inhibition [3], serum immune and antioxidant enzyme activity improvement [4], and treatment of Alzheimer’s disease [5] and liver disease [6]. Emodin also has industrial applications, such as food additives, dyes, papermaking, and cosmetics [7]. Emodin has mainly been isolated from plants, fungi, and even some animals [8]. However, thus far, most have been obtained from herbs such as rheum palmatum [9]. The development of emodin was hardly influenced by the long planting and growth cycle time cost of rhubarb, the phenomenon of soil obstacle to continuous cropping, the high cost of prevention of diseases and insect pests, and the low efficient of extraction. Therefore, it is urgent to produce emodin in a more efficient and inexpensive way.

**Figure 1:**
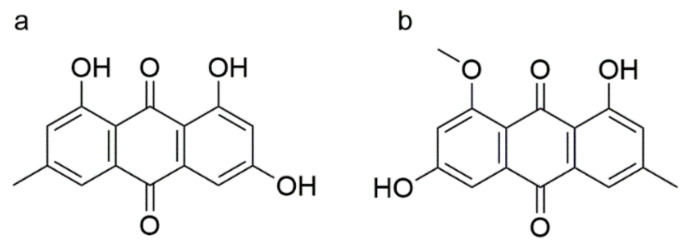
The chemical structures of emodin (**a**) and questin (**b**).

Emodin can be biosynthesized by the fungi from the genera of *Aspergillus* and *Penicillium*. A high emodin production fungus *Aspergillus ochraceus* was screened by Lu et al. to produce emodin at a preparation scale with the yield of 1.453 mg/L, which was higher than the yield isolated from medicinal materials [10]. In order to seek higher emodin production, the research of marine fungi has opened up a new way due to the unique living environment characterized by high salinity and hypertonic, weak nutrition, and alkalescence [11]. Emodin (Figure 1a) and questin (Figure 1b) are anthraquinone compounds isolated and identified from marine-derived filamentous fungus *Aspergillus favipes* HN4-13. Questin shows favorable antimicrobial effects, which can be converted into emodin by demethylation reaction in high ratio. Guo et al. optimized the production of questin by *A. favipes* HN4-13; however, the production is low, and only the predicted diameter of inhibitory zone against *Vibrio harveyi* of questin is used as the evaluation index [12,13].

Intracellular inorganic polyphosphate concentration is essential for the survival, growth, stress response, biofilm synthesis, mycelial morphology, and other functions of microorganisms. In this work, single-factor experiments, Plaekett–Burman design (P-B design), central composite design (CCD), and strategy of phosphorus supplementation were adopted to optimize the culture medium and parameters for the production of emodin through a marine-derived fungus *A*. *favipes* HN4-13. Metabolic parameters (dry cell weight (DCW), residual KH_2_PO_4_, emodin production, and questin production) and transcript levels of critical biosynthesis cluster genes (*emo*A-K) were detected with the optimal fermentation conditions. The effect of different phosphorus source on the fungus morphology was firstly investigated, and the results suggested that KH_2_PO_4_ was positive for emodin and questin production. Furthermore, the conversion from questin to emodin by demethylation reaction has an efficient conversion rate (92.64 ± 2.10%, 6 h). The information obtained would be useful for improving emodin production in submerged fermentation.

## 2. Results

### 2.1. Single-Factor Investigation

The effects of different fermentation conditions, such as carbon sources, nitrogen sources, inorganic salts, inoculum size, pH, and culture days, were examined according to the production of emodin by *A. flavipes* HN4-13. The single-factor investigation results showed that 50 g/L soluble starch, 10 g/L yeast extract paste, 30 g/L seawater salt, 1 g/L KH_2_PO_4_, 0.5 g/L MgSO_4_·7H_2_O, 0.01 g/L FeSO_4_·7H_2_O (pH 5), 18% inoculum size, and 7-day culture are favorable for the production of emodin.

### 2.2. Screening of Significant Factors through P-B Design

From the single factor optimization of fermentation medium, we found that the components of culture medium had obvious influence on emodin biosynthesis. In order to further explore the effect of the concentration of medium components on the biosynthesis of emodin, we conducted the P-B design using the statistical software Design Expert 12.0 to explore the factors that significantly affected the synthesis of emodin in the medium components (including soluble starch, yeast extract, KH_2_PO_4_, MgSO_4_·7H_2_O, and FeSO_4_·7H_2_O).

In accordance with the experimental design matrix, 12 groups of experiments were carried out, with three replicates in each group, and the average production of emodin in each group was taken as the response value. The experimental results and ANOVA analysis results are shown in Tables S1–S4. The level of significance follows the order soluble starch > KH_2_PO_4_ > FeSO_4_·7H_2_O > MgSO_4_·7H_2_O > yeast extract paste. *p*-values of soluble starch, KH_2_PO_4_, and FeSO_4_·7H_2_O were significant variables (*p*-value ≤ 0.05). The increased amount of KH_2_PO_4_ had a positive effect on the production of emodin, while soluble starch and FeSO_4_·7H_2_O had negative effects.

### 2.3. Single-Factor Gradient Experiment Investigation

On the basis of P-B design experimental results, we selected soluble starch, KH_2_PO_4_, and FeSO_4_·7H_2_O for response surface analysis. In order to obtain more accurate results of response surface analysis, one should first determine the center point close to the optimal region. When determining the center point, we used the single factor gradient change to determine the center point.

**Figure 2:**
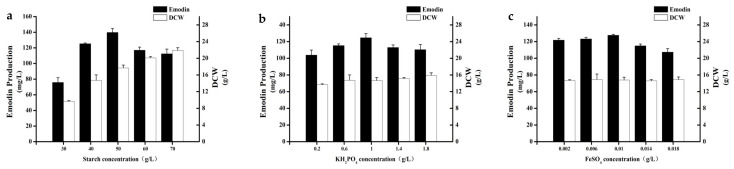
(**a**) Effect of soluble starch concentration on cell growth and emodin production. (**b**) Effect of KH_2_PO_4_ concentration on cell growth and emodin production. (**c**) Effect of FeSO_4_·7H_2_O concentration on cell growth and emodin production.

The concentrations of the other components in the basal medium were kept constant, except for the three factors. Five different gradients of the three changing factors were selected for experiments, The concentrations of soluble starch were set as follows: 30 g/L, 40 g/L, 50 g/L, 60 g/L, and 70 g/L. The concentrations of KH_2_PO_4_ were set as follows: 0.2 g/L, 0.6 g/L, 1 g/L, 1.4 g/L, and 1.8 g/L. The concentrations of FeSO_4_·7H_2_O were set as follows: 0.002 g/L, 0.006 g/L, 0.01 g/L, 0.014 g/L, and 0.018 g/L. The experimental results are shown in Figure 2. The optimum concentration was found to be soluble starch 50 g/L, KH_2_PO_4_ 1 g/L, FeSO_4_·7H_2_O 0.01 g/L. Therefore, this combination was finally selected as the central point for the next response surface optimization experiment.

### 2.4. Optimization of Significant Factors through CCD

On the basis of the P-B design and single-factor gradient experiment results, we applied CCD to further optimize the levels of the three significant variables. Furthermore, the response surface mathematical (RSM) model was established to optimize the concentration of three key factors affecting the biosynthesis of emodin. The coded value for soluble starch, KH_2_PO_4_, and FeSO_4_·7H_2_O in the CCD are shown in Table 1, and the matrices and results of CCD are shown in Table 2. On the basis of the parameter estimates of CCD, the RSM was able to provide the empirical relationships between response variable and independent variables. Therefore, the following second-order polynomial equation was established:

*Y* = 124.15 + 17.51*A* + 2.19*B*−1.79*C* + 7.16*AB* + 2.78*AC*−3.22*BC*−10.25*A*^2^−21.47*B*^2^−13.5*C*^2^.

Table 3 shows the results of ANOVA. The *p*-value of Model < 0.0001 and lack of fit (0.0007) revealed that the model was significant. *A*, *AB*, *A*^2^, *B*^2^, and *C*^2^ were the significant terms of the model (*p* < 0.05). Moreover, the determination *R*^2^ (0.9665) showed that 96.65% of the experimental results can be explained by the model, and the equation fit well. The relative deviation *CV* = 7.5% was within a reasonable range. The adjustment decision coefficient Adj*R*^2^ = 0.9364 was close to the decision coefficient, which further proved the reliability of the model.

**Figure 3:**
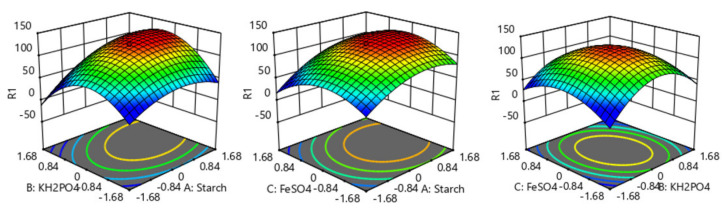
Response surface plot for the effects of two various factors on emodin production and their interactions.

Figure 3 shows the interactive effects of two variables on emodin production. It can be seen from the figure that each fitted surface had a maximum value, which showed that there was an optimal combination of these different factors that was beneficial to emodin biosynthesis. According to such a surface, the production of emodin can be predicted under different conditions. Therefore, the best concentration of culture components was obtained by fitting analysis with Design Expert software as follows: When soluble starch, KH_2_PO_4_, and FeSO_4_·7H_2_O were 59.3 g/L (coded value 0.926), 1.04 g/L (coded value 0.205), and 0.01 g/L (coded value 0.005), respectively, the predicted maximal production of emodin (132.47 mg/L) was achieved by the model.

### 2.5. Model Validation

Finally, the optimized fermentation parameters and medium composition for emodin production in a 250-mL conical flask: 59.3 g/L soluble starch, 10 g/L yeast extract paste, 30 g/L seawater salt, 1.04 g/L KH_2_PO_4_, 0.05 g/L MgSO_4_·7H_2_O, 0.01 g/L FeSO_4_·7H_2_O, pH 5, 18% inoculum size, and shaking at 32 °C and 160 rpm/min for 7 days. Under these conditions, the production of emodin was achieved as 132.40 ± 3.09 mg/L (*n* = 9, Table 4), which showed no significant difference when compared with the predicted value (132.47 mg/L).

This showed that the prediction model was reliable and can enhance the production of emodin. Meanwhile, the production of emodin in the optimized conditions was 11-fold higher than that of the initial medium.

### 2.6. Demethylation of Questin

**Figure 4:**
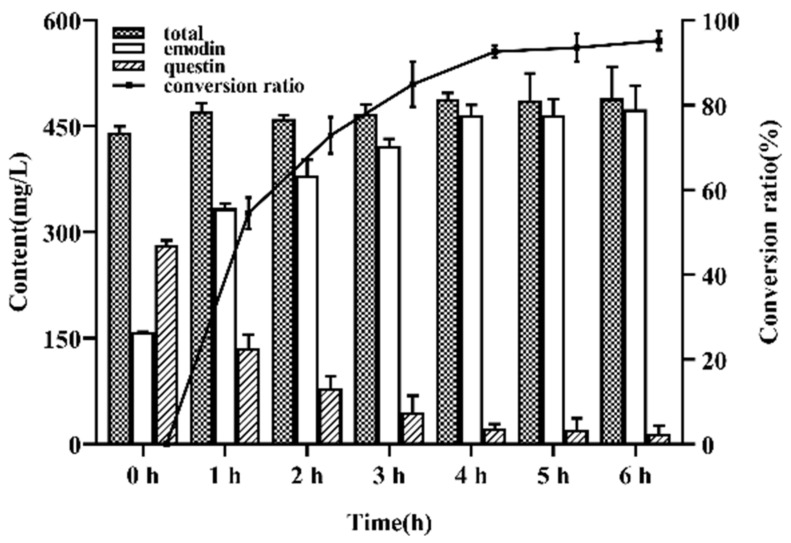
Conversion rates of demethylation conversion of questin.

Part of the pretreated fermented extract was taken, and questin was converted into emodin by demethylation (Figure 4). When the reaction time was 4 h, the conversion of questin to emodin almost reached the equilibrium, and the conversion rate was 92.64 ± 2.10%.

### 2.7. Effects of Proposal of Phosphorus Supplement Strategy on Presumed Metabolic Pathway

Excessive phosphate is unfavorable to production, but lack of phosphate will affect the growth of microorganisms. Phosphate concentration in the range of 0.3 to 300 mmol/L will not inhibit cell growth, while inorganic phosphorus in excess of 10 mmol/L will inhibit the biosynthesis of many antibiotics and other secondary metabolites. Inorganic phosphorus promotes primary metabolism and inhibits secondary metabolism, while phosphorus limits the synthesis of secondary metabolite synthesis inducers. Excessive phosphorus inhibits the formation of secondary metabolite precursors [14].

**Figure 5:**
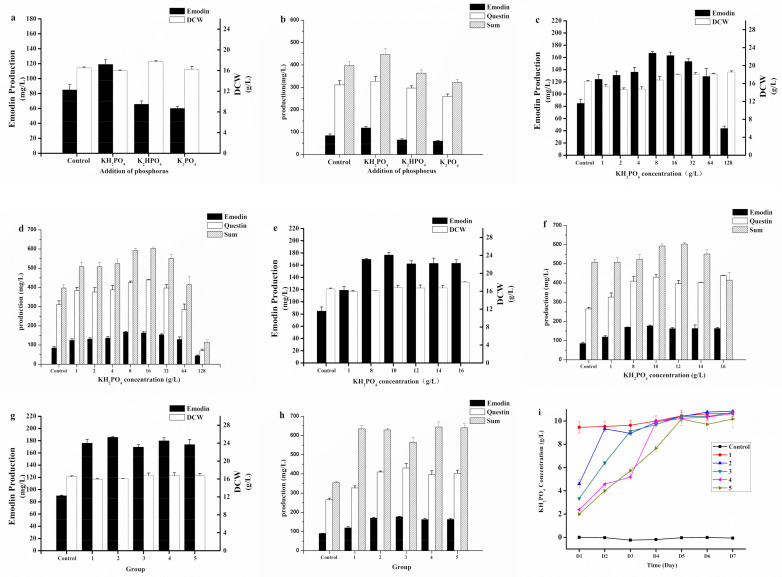
Emodin production, DCW, questin production, the sum of emodin and questin production at 168 h (160 rpm, 32 °C) by *A. flavipes* HN4-13. (**a**,**b**) Effect of different inorganic polyphosphate. (**c**–**f**) Effect of different KH_2_PO_4_ concentrations. (**g,h**) Effect of different KH_2_PO_4_ supplementation strategy. (**i**) Residual phosphorus with different KH_2_PO_4_ supplementation strategy (measured before supplementation).

On the basis of P-B design experimental results, we found that the increased amount of KH_2_PO_4_ had a positive effect on the production of emodin, and 1 g/L KH_2_PO_4_ (7.348 mmol/L KH_2_PO_4_, 1.672 mmol/L P) was in a reasonable concentration range. Control medium was 59.3 g/L soluble starch, 10 g/L yeast extract paste, 30 g/L seawater salt, 0.05 g/L MgSO_4_·7H_2_O, 0.01 g/L FeSO_4_·7H_2_O, pH 5, 18% inoculum size, and shaking at 32 °C and 160 rpm/min for 7 days; in order to investigate different forms of phosphate, we added the same mol of phosphate to the medium as other parameters in order for it to be kept constant. As shown in Figure 5a,b, KH_2_PO_4_ was found to be the best phosphorus source for *A. favipes* HN4-13. Since the effect of KH_2_PO_4_ was obvious, the next step was to try to increase the concentration of KH_2_PO_4_ substantially. According to the experimental results (Figure 5c,d), interestingly, biomass was not affected by increasing concentrations of KH_2_PO_4_. Emodin synthesis was not inhibited until the concentration reached 128 g/L. As shown in Figure 5e,f, 10g/L KH_2_PO_4_ was selected as the best concentration, and the production of emodin was 176.64 ± 4.03 mg/L.

On this basis, we put forward the inorganic polyphosphate supplement strategy. The strategy of KH_2_PO_4_ supplementation is shown in Table 5, and the concentration of KH_2_PO_4_ in the medium was also determined while the production of emodin was determined. As shown in Figure 5g,h, the experimental results showed that different strategies had a miniscule difference on the production of emodin. Group 2 was the better strategy, and the production of emodin was 185.56 ± 4.39 mg/L. Therefore, the addition of 10 g/L KH_2_PO_4_ at the early stage of culture was the most convenient, and the addition or not of KH_2_PO_4_ had a tremendous influence on the production.

### 2.8. Transcriptional Levels of Key Genes in Polyketide Pathway

**Figure 6:**
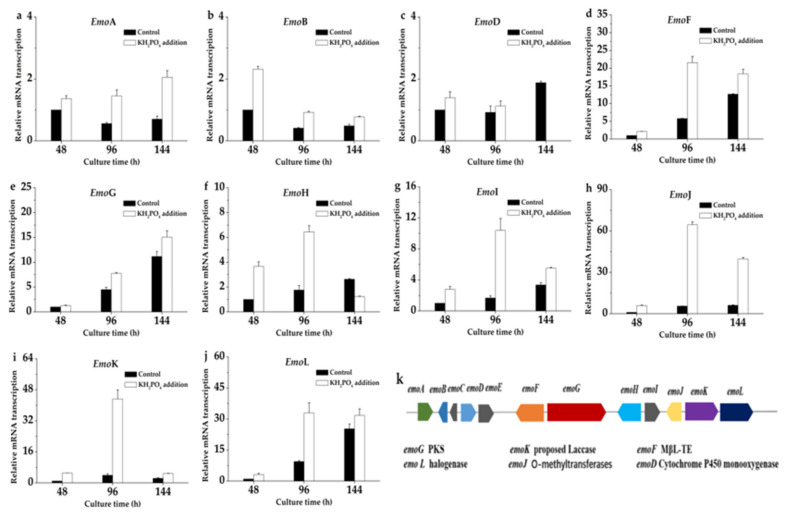
Response of transcriptional level of genes related to emodin biosynthesis to control at 48, 96, and 144 h. (**a**) EmoA relative mRNA transcription. (**b**) EmoB relative mRNA transcription. (**c**) EmoD relative mRNA transcription. (**d**) EmoF relative mRNA transcription. (**e**) EmoG relative mRNA transcription. (**f**) EmoH relative mRNA transcription. (**g**) EmoI relative mRNA transcription. (**h**) EmoJ relative mRNA transcription. (**i**) EmoK relative mRNA transcription. (**j**) EmoL relative mRNA transcription. (**k**) Proposed biosybthesis cluster of emodin in A. flavipes HN4-13.

Sun et al. reconstituted a biosynthetic pathway of emodin in Saccharomyces cerevisiae and confirmed that the biosynthetic pathway is polyketide pathway [15]. To provide an insight into the probable mechanism underlying the effect of KH_2_PO_4_ adding on emodin production, we detected expression levels of 12 genes emoA, emoB, emoC, emoD, emoE, emoF, emoG, emoH, emoI, emoJ, emoK, and emoL of polyketide pathway by qRT-PCR. As shown in Figure 6, the transcriptional levels of these genes were investigated at 48, 96, and 144h in KH_2_PO_4_ adding experience.

As shown in Figure 6c, the transcription level of emoD (cytochrome P450 monooxygenase) was slightly increased with the 10 g/L KH_2_PO_4_ addition, and the transcriptional level of emoD was increased by about 1.39-fold at 48 h and 1.22-fold at 96 h, but it was interesting that the transcriptional level of emoD was not detected at 144 h. The transcription level of emoF was significantly increased with the 10 g/L KH_2_PO_4_ addition, and the transcription level of emoF was increased by about 2.1-fold at 48 h, 3.7-fold at 96 h, and 1.45-fold at 144 h (Figure 6d). The effects of KH_2_PO_4_ addition on emoG, emoJ, emoK, and emoL transcription were similar to emoF, being increased 1.27-fold at 48 h, 1.72-fold at 96 h, and 1.35-fold at 144 h (Figure 6e); 5.91-fold at 48 h, 11.68-fold at 96 h, and 6.45-fold at 144 h (Figure 6h); 5.08-fold at 48 h, 11.02-fold at 96 h, and 2.06-fold at 144 h (Figure 6i); 3.19-fold at 48 h, 3.49-fold at 96 h, and 1.26-fold at 144 h (Figure 6j), respectively. In addition, except that emoC and emoE were not detected, emoA (Figure 6a), emoB (Figure 6b), emoH (Figure 6f), and emoI (Figure 6g) in the gene cluster were improved to a certain extent. These results indicated the key genes of polyketide pathway were upregulated with the addition of KH_2_PO_4_.

Many previous studies have reported that the upregulated expression of biosynthetic gene clusters can improve the development of fungi and the biosynthesis of secondary metabolism. Michaela Králová et al. overexpressed the Trp-related genes in Claviceps purpurea, which led to increased ergot alkaloid production [16]. In this work, the addition of KH_2_PO_4_ resulted in the improvement of transcription level of key enzyme genes in polyketide pathway and emodin production, which firstly implied that KH_2_PO_4_ could promote expression the key enzyme genes of biosynthesis of emodin in the fermentation of A. flavipes HN4-13. Therefore, the results of qRT-PCR indicated that the addition of KH_2_PO_4_ improved the transcription levels of specific genes of polyketide pathway to improve the production of emodin.

### 2.9. Effect of KH_2_PO_4_ on Hypha Morphology

The effects of KH_2_PO_4_ on the fermentation process of A. flavipes HN4-13 were determined by comparing the emodin, questin production, and hypha morphology of control group; 10 g/L KH_2_PO_4_ addition group; and 10 g/L KH_2_PO_4_ addition group (grinded at 48 h).

**Figure 7:**
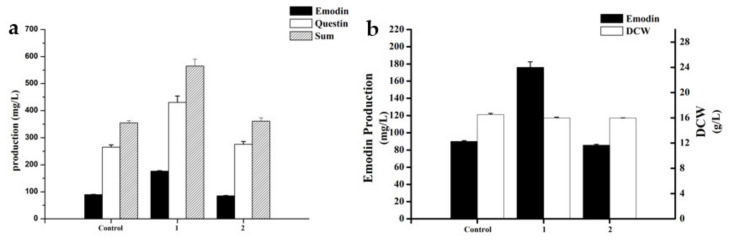
(**a**) Effect of different treatment methods on DCW and emodin production. (**b**) Effect of different treatment methods on emodin and questin production.

As shown in Figure 7, 1 represented the 10 g/L KH_2_PO_4_ addition group (10 g/L), and 2 represented the 10 g/L KH_2_PO_4_ addition group (grinded at 48 h). The experimental results showed that the production of emodin and questin increased by 118.69% when 10 g/L KH_2_PO_4_ was added compared with the non-addition group. However, when 10 g/L KH_2_PO_4_ was added with grinding at 48 h, the addition of KH_2_PO_4_ did not have a production-promoting effect.

**Figure 8:**
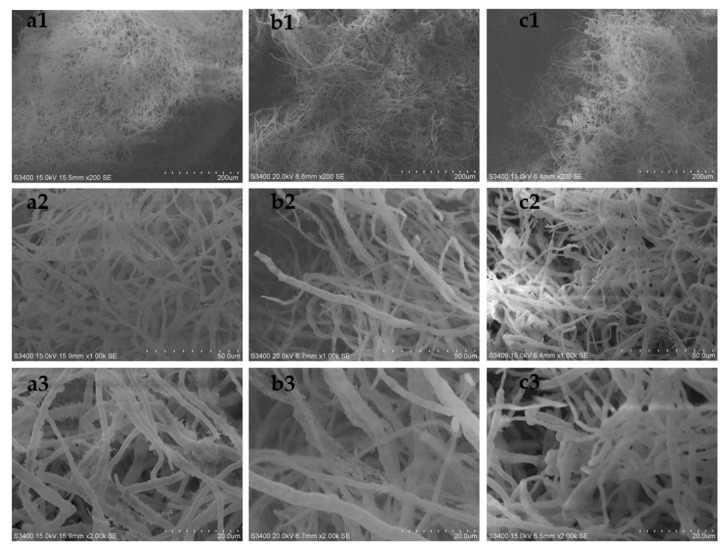
(**a1**–**a3**) Hypha morphology of the control group under 200×, 1000×, and 2000×, respectively. (**b1**–**b3**) Hypha morphology of the 10g/L K_2_HPO_4_ addition group under 200×, 1000×, and 2000×, respectively. (**c1**–**c3**) Hypha morphology of the 10g/L K_2_HPO_4_ addition group (grinded at 48 h) under 200×, 1000×, and 2000×, respectively.

In order to explore its mechanism, we observed the morphological differences among the above three groups by scanning electron microscopy. The results are shown in Figure 8 (the seventh day of fermentation). On the seventh day of fermentation, the hypha morphology of the KH_2_PO_4_ addition group (Figure 8a) was significantly more robust than that of the control group (Figure 8b) and the grinding group (Figure 8c).

## 3. Materials and Methods

### 3.1. Microorganism

*A. favipes* HN4-13 strain (CCTCC no. AF2,015,022) was maintained on an agar plate (4% glucose, 1% peptone, 2.5% agar, and 3% seawater salt were dissolved deionized water) and stored at 4 °C.

### 3.2. Fermentation Conditions

*A. favipes* HN4-13 was cultured on an agar plate at 28 °C for 5 days. A square ager piece (size of 10 × 10 mm) was taken out the plate and transferred to a 250 mL shake flask with 100 mL seed medium (40 g/L glucose, 10 g/L peptone, 30 g/L seawater salt dissolved deionized water) for culture at 180 rpm, 28 °C for 24 h. Subsequently, 9 mL (18% inoculum size) of seed medium was inoculated into a 250-mL Erlenmeyer flask with 50 mL fermentation medium (50 g/L soluble starch, 10 g/L yeast extract paste, 30 g/L seawater salt, 1 g/L KH_2_PO_4_, 0.5 g/L MgSO_4_·7H_2_O, 0.01 g/L FeSO_4_·7H_2_O) at 160 rpm, 32 °C for 168 h.

### 3.3. Preparation of Extracts

In order to separate the broths from mycelium, we separated the fermentation broth by Buchner funnel. In order to improve the extraction efficiency, the harvested fermentation broth was crushed with ultrasonic waves and extracted with twice the volume of ethyl acetate (EtOAc) for three times to obtain ethyl acetate solution containing the target product. Then, the active extracts were obtained through concentrating the EtOAc solution by reduced pressure rotary evaporator and dissolving into 25 mL CH_3_OH. Finally, it was filtered by 0.22 μm microporous membrane and stored at 4 °C.

### 3.4. Single-Factor Experiments

We obtained an initial culture medium through screening (40 g/L glucose, 10 g/L peptone, 30 g/L seawater salt, 1 g/L KH_2_PO_4_, 0.5 g/L MgSO_4_·7H_2_O, 0.01 g/L FeSO_4_·7H_2_O). In order to screen the carbon source, we used soluble starch, sucrose, maltose, or mannitol instead of glucose and the nitrogen source in the medium. In order to screen the nitrogen source, we replaced peptone with yeast extract paste, yeast extract powder, soybean meal, corn flour, or ammonium sulfate, while the carbon source was kept unchanged. Deionized water containing 3% sea salt was used to replace naturally aged seawater.

Seed culture time (12 h, 24 h, 36 h, 48 h, 60 h, 72 h), inoculum size (6%, 10%, 14%, 18%, 22%), pH (4, 5, 6, 7, 8), temperature (26 °C, 28 °C, 30 °C, 32 °C, 34 °C), rotation speed (120 rpm/min, 140 rpm/min, 160 rpm/min, 180 rpm/min, 200 rpm/min), and fermentation time (72 h, 96 h, 120 h, 144 h, 168 h, 192 h) were also determined by screening, with other parameters kept constant. The best single factors or levels were determined according to the production of emodin.

### 3.5. Placket–Burman Design

The fermentation variables having significant influence on emodin production were optimized by Placket–Burman design (P-B design). According to the results of Placket–Burman design, 3 main factors could be selected from 5 variables using 12 experiments. The Placket–Burman design experiments (Tables S1 and S2) were designed using Design Expert 12 software (Stat-Ease, Minneapolis, MN, USA), and each variable had two levels coded as −1 and +1. The regression coefficients were determined by testing. It is considered that the *p*-value of variable is not more than 0.05, which is of great significance to the production or yield of emodin.

### 3.6. Single-Factor Gradient Experiment

Through the Placket–Burman design experiment, we were able to select three main factors (*p*-value ≤ 0.05). Except for the three factors, the concentration of other components in the medium was kept constant. As the basis of central composite design experiment, the concentration gradient experiment was carried out on the selected three main influencing factors to preliminarily determine the more suitable concentration.

### 3.7. Central Composite Design

On the basis of single-factor gradient experiment, we were able to carry out the central composite design (CCD design). CCD is one of response surface methods to optimize significant variables, which used Design Expert 12 to establish the model for analyzing the experimental data [17]. CCD consisted of 20 experimental points, of which 14 factorial points and 6 central points are composed. 

Soluble starch (A), KH_2_PO_4_ (B), and FeSO_4_·7H_2_O (C) were chosen as independent variables; the concentration of other components in the medium was kept constant; and the dependent variable (Y, mg/L) was the production of emodin. The coded and values of soluble starch (A), KH_2_PO_4_ (B), and FeSO_4_·7H_2_O (C) are shown in Table 1 and Table 2**.** The goal of optimization is to achieve the maximum production of emodin by using design expert 12. The results of CCD were conformed according to the following second-order polynomial equation:

$$Y=\beta_{0}+\sum_{i=1}^{3}\beta_{i}X_{i}+\sum_{i=1}^{3}\beta_{ii}X_{i}^{2}+\sum_{i=1}^{2}\sum_{j=i+1}^{3}\beta_{ij}X_{i}X_{j},$$

where *Y* represents the predicted response; *β_0_*, *β_i_*, *β_ii_*, and *β_ij_* represent the constant coefficients; and *X_i_* and *X_j_* represent the independent variables.

### 3.8. Analysis of the Production of Emodin

The emodin standard substance was dissolved in methanol and determined by HPLC instrument (Agilent Technologies 1260 Infinity, Santa Clara, CA, USA) on an ODS column (Eclipse Plus C18, 4.6 mm × 250 mm, 5 μm, 0.8 mL/min) to obtain a standard curve. The peak areas of emodin in active extracts were determined using HPLC. The peak areas were converted to the concentrations of emodin through the standard curve. The concentrations of the standard solutions ranged from 31.25 to 500 mg/L, and the ultraviolet detection was at 310 nm. The gradient elution procedure (mobile phase) consisting of water and methanol was as follows: 0.00–13.00 min 10–100% (*v*/*v*) CH_3_OH, 13.01–18.00 min 100% CH_3_OH, 18.01–18.50 min 100%–10% CH_3_OH, and 18.51–23.00 min 10% CH_3_OH. Then, the yield of emodin under different conditions was calculated using the linear equation: *Y* = 5.326*X* + 9.275 (*R*^2^ = 0.9992). *X* is the peak area of emodin, and *Y* is the concentration of emodin.

### 3.9. Demethylation of Questin

Questin can be converted into emodin by demethylation reaction. The method was as follows: the active extracts were obtained through concentrating the EtOAc solution by using reduced pressure rotary evaporator, which dissolved in 60 mL acetic acid, adding 25 mL HBr, refluxing in 85 °C water bath heating for 6 h. Then, the organic solvent was recovered by rotary evaporator, redissolved by methanol, and analyzed by HPLC.

### 3.10. Proposal of Inorganic Polyphosphate Supplement Strategy

According to previous experiments, the addition of KH_2_PO_4_ can enhance the production of emodin. A different inorganic polyphosphate supplementation strategy was proposed to pursue higher production of emodin. Consequently, according to the production of emodin, we obtained the best supplement strategy. In order to study the content of phosphorus in different groups of fermentation broth, we used molybdenum blue spectrophotometry to determine phosphorus in fermentation broth.

### 3.11. The Informations of Key Gene of emodin Biosynthesis Gene Cluster

The emodin biosnthesis gene cluster from *A. favipes* HN4-13 was deduced, including *emo*A, *emo*B, *emo*C, *emo*D (Cytochrome P450 monooxygenase), *emo*E, *emo*F (MβL-TE), *emo*G (PKS), *emo*H, *emo*I, *emo*J (*O*-methyltransferases), *emo*K (proposed Laccase), and *emo*L (halogenase). According to the information from NCBI, the results of *A. favipes* HN4-13 homology correlation of these genes were shown in Table S5.

### 3.12. Measurement of Key Enzyme Gene Expressions by Real-Time Quantitative PCR (qRT-PCR)

The genome of *A. favipes* HN4-13 was utilized to design the primers of internal reference gene. The total RNA of *A. favipes* HN4-13 was extracted by extracting solution (TriZol) and using a spectrophotometer (A260/280) to determine RNA concentration. The experimental procedure of real-time quantitative PCR was used with the improved method of Li et al. [18]. The starting materials were total RNA and the PremixScript™ Reagent Kit (Takara), which were used for reverse transcription. According to manufacturer’s procedure, the transcriptional levels of *emo*A, *emo*B, *emo*C, *emo*D, *emo*E, *emo*F, *emoG*, *emo*H, *emo*I, *emo*J, *emo*K, *emo*L, and actin were determined by qRT-PCR carried out on a Premix Ex Taq™ II (Takara).

The process of qRT-PCR was as follows: predenature at 95 °C for 10 min. Then, amplification occurred in two steps: 20 s at 95 °C for denature, 30 s at 58 °C for annealing, and 25 s at 72 °C for extension for 45 cycles. Finally, the expression of the gene relative to the internal reference gene was calculated using the dissolution curve *Ct*:

ΔΔ*Ct* = Δ*Ct* (pilot sample) − Δ*Ct* (reference sample)

Δ*Ct* (pilot sample) = *Ct* (pilot sample, target gene) −*Ct* (pilot sample, internal reference gene)

Δ*Ct* (reference sample) = *Ct* (reference sample, target gene) −*Ct* (reference sample, internal reference gene)

The expression level of that reference sample is taken as 1. The value of 2^−ΔΔ*Ct*^ is the gene expression of the sample.

The sequences of forward/reverse primers of the *A. favipes* HN4-13 *emo*A, *emo*B, *emo*C, *emo*D, *emo*E, *emo*F, *emo*G, *emo*H, *emo*I, *emo*J, *emo*K, and *emo*L are listed in Table S6.

### 3.13. Observation Method of A. favipes HN4-13 Morphology

The morphology of *A. favipes* HN4-13 was observed by scanning electron microscope. The fermentation broth was centrifuged in a 3500 r/min centrifuge for 10 min. After the samples were collected, they were rinsed with 0.1 mol/L PBS buffer, and then centrifuged in a 3500 r/min centrifuge for 10 min. The experiments were repeated three times. The samples were fixed in the dark with 2.5% glutaraldehyde fixative for 2 h, rinsed with 0.1 M PBS buffer, and centrifuged again in a 3500 r/min centrifuge for 10 min for four times. The samples were dehydrated step by step with 50%, 75%, 95%, and 100% ethanol, and then critically dried with carbon dioxide. After ion spraying, the samples were observed by scanning electron microscope (Hitachi S3400N), and the images were taken under 200×, 1000×, and 2000×.

## 4. Conclusions

Most of the functional active natural products come from sponges, microorganisms, stinging animals, etc. [19,20,21]. Marine-derived microorganisms are undeveloped wild flora for discovery of effective active compounds. The low yield of secondary metabolites has been limited the source application and development. Therefore, the source problem of natural products can be well solved by optimizing the fermentation process and then conducting the expanded culture [22]. The interactions among fermentation conditions are complicated, and depend on many variables. The nutrients (including carbon source, nitrogen source, and ion type) in the fermentation medium can not only affect the spore germination, cell growth, and development of microorganisms, but also affect the synthesis of secondary metabolites and active compounds. Therefore, the use of statistical methods can optimize microbial culture media and fermentation parameters to optimize biological processes. Dinarvand et al. optimized the fermentation medium using the CCD response surface method to obtain the medium component with the highest yield of inulinase and invertase synthesized by *A*. *niger* ATCC 20611 [23].

In the present study, the fermentation conditions for emodin production from *A. favipes* HN4-13 were optimized by RSM, which were divided into five steps: Firstly, the basic fermentation conditions of *A. favipes* HN4-13 were determined by single-factor experiment. Secondly, the influencing factors were investigated by P-B design, where it was identified that soluble starch, KH_2_PO_4_, and FeSO_4_·7H_2_O were the significant factors. Thirdly, the central point close to the optimal region was determined through the single-factor gradient experimental study. Fourth, tri-factors and five-level CCD were adopted to optimize the levels of significant factors. Lastly, the reliability of the regression equation and the validity of the statistical method were verified by experiments. In conclusion, the optimized fermentation conditions for the production of emodin through *A. favipes* HN4-13 in a 250-mL conical flask with 50 mL of medium were 59.3 g/L soluble starch, 10 g/L yeast extract paste, 30 g/L seawater salt, 1.04 g/L KH_2_PO_4_, 0.05 g/L MgSO_4_·7H_2_O, 0.01 g/L FeSO_4_·7H_2_O, seed culture 24 h, pH 5, inoculum size 18%, culture temperature 32 °C, and shaking 160 rpm/min for 7 days. The production of emodin achieved 132.40 ± 3.09 mg/L that was 11-fold higher than that of the initial medium; meanwhile, production of by-product questin can achieve 390.58 ± 9.49 mg/L.

Interestingly, the strain showed strong tolerance to KH_2_PO_4_. Therefore, a strategy of KH_2_PO_4_ adding was proposed to enhance the production of emodin to 185.56 ± 4.39 mg/L, which was 15.87-fold higher than that of the initial medium. Meanwhile, the production of by-product questin can achieve 443.33 ± 4.83 mg/L, which can be converted into emodin by demethylation reaction (conversion ratio 92.64% ± 2.10%, 4 h). The final emodin production can reach 602.29 ± 4.47 mg/L, which is 54.73-fold of the initial production. Results showed that KH_2_PO_4_ could improve the expression of emodin synthesis genes by measuring their transcriptional level in control group and KH_2_PO_4_ added group. Furthermore, KH_2_PO_4_ could make the hyphae of the strain robust, and the grinding group’s results further confirmed that KH_2_PO_4_ changed the morphology of *A. favipes* HN4-13 and improved the secondary metabolism production. The effect of such a high concentration of KH_2_PO_4_ on fungi has not been reported. However, the specific mechanism of KH_2_PO_4_ activating emodin synthesis gene cluster had not yet been determined, which would be studies in following research. Above results showed that *A. favipes* HN4-13 was expected to be the source for large-scale production of emodin in submerged fermentation. The construction of the emodin biosynthetic production platform enables efficient and environmentally friendly scale-up production, which facilitates the conservation of wild plant resources. This work provided a new approach for further enhancement and large-scale fermentation of emodin.

## Figures and Tables

**Table 1:** The coded value for each single variable in the CCD.

Level	X_1_ (Soluble Starch)	X_2_ (KH_2_PO_4_)	X_3_ (FeSO_4_·7H_2_O)
−1.68179	33.2 g/L	0.664 g/L	0.00664 g/L
−1	40 g/L	0.8 g/L	0.008 g/L
0	50 g/L	1 g/L	0.01 g/L
+1	60 g/L	1.2 g/L	0.012 g/L
+1.68179	66.8 g/L	1.336 g/L	0.01336 g/L

**Table 2:** The production of emodin in the CCD experiment.

No.	Variable			Emodin (mg/L)
	A (Soluble Starch)	B (KH_2_PO_4_)	C (FeSO_4_·7H_2_O)	
1	−1	−1	−1	60.67 ± 1.75
2	1	−1	−1	85.76 ± 1.14
3	−1	1	−1	57.77 ± 2.44
4	1	1	−1	108.04 ± 6.27
5	−1	−1	1	64.98 ± 2.37
6	1	−1	1	97.73 ± 3.02
7	−1	1	1	45.73 ± 2.30
8	1	1	1	110.61 ± 6.97
9	−1.68179	0	0	75.52 ± 6.14
10	1.68179	0	0	114.81 ± 3.14
11	0	−1.68179	0	58.42 ± 1.45
12	0	1.68179	0	68.47 ± 1.82
13	0	0	−1.68179	95.30 ± 5.59
14	0	0	1.68179	76.70 ± 6.07
15	0	0	0	123.54 ± 2.51
16	0	0	0	124.22 ± 4.89
17	0	0	0	125.12 ± 1.98
18	0	0	0	126.53 ± 3.22
19	0	0	0	121.58 ± 2.21
20	0	0	0	123.88 ± 2.88

**Table 3:** Analysis of variance for the relationships between response variable and independent variables.

Source	DF	Sum of Square	Mean Square	*F*-Value	*p*-Value	Sig.
Model	9	14,110.55	1567.84	32.08	<0.0001	**
A	1	4185.04	4185.04	85.64	<0.0001	**
B	1	65.5	65.50	1.34	0.2739	
C	1	43.79	43.79	0.8960	0.3662	
A × B	1	410.47	410.47	8.40	0.0159	*
A × C	1	61.95	61.95	1.27	0.2865	
B × C	1	82.83	82.83	1.69	0.2221	
A × A	1	1515.51	1515.51	31.01	0.0002	**
B × B	1	6642.44	6642.44	135.92	<0.0001	**
C × C	1	2624.66	2624.66	53.71	<0.0001	**
Error	10	488.70	48.87			
Total	19	14,599.25				

** *p* < 0.01, * 0.01 < *p* < 0.05.

**Table 4:** Model verification of emodin production after medium optimization.

	No.1 (mg/L)	No.2 (mg/L)	No.3 (mg/L)	Average (mg/L)
Batch 1	135.72	132.53	133.32	133.86
Batch 2	136.76	124.30	131.33	130.80
Batch 3	132.63	129.98	134.99	132.53
Average (mg/L)	132.40			

**Table 5:** The strategy of KH_2_PO_4_ supplementation.

Group	Day 1	Day 2	Day 3	Day 4	Day 5	Day 6	Day 7	Total (g/L)
1	10	0	0	0	0	0	0	10
2	5	5	0	0	0	0	0	10
3	3.33	3.33	3.33	0	0	0	0	10
4	2.5	2.5	2.5	2.5	0	0	0	10
5	2	2	2	2	2	0	0	10

## Supplementary Materials

The following are available online at https://www.mdpi.com/article/10.3390/md19080421/s1, Table S1: The (+) and (−) value for each single variable in the P-B design. Table S2: The production and yield of emodin in the P-B design. Table S3: ANOVA for P-B design experiments of emodin production. Table S4. ANOVA for P-B design experiments of emodin yield. Table S5: The homology correlation of these genes between *A. favipes HN4-13* and the information from NCBI. Table S6: Sequences of primer pairs for quantitative real-time RT-PCR (qRT-PCR) assay.
