# Supplementary material for: Enhancement of Emodin Production by Medium Optimization and KH2PO4 Supplementation in Submerged Fermentation of Marine-Derived Aspergillus favipes HN4-13
Source: Mar Drugs. 2021 Jul 26;19(8):421. doi: 10.3390/md19080421 (PMC8398268; doi:10.3390/md19080421)
Supplement: Supplementary file 1 [file marinedrugs-19-00421-s001.zip › marinedrugs-1275131-supplementary.pdf]

Enhancement of Emodin Production by Medium Optimization and KH_2PO_4 Supplementation in Submerged Fermentation of Marine derived *Aspergillus favipes* HN4-13

Xiaohan Qiu, Lizhi Gong, Xiujuan Xin, Faliang An*

Table S1. The (+) and (−) value for each single variable in the P-B design.

Level	X_1 (Soluble starch)	X_2 (yeast extract paste)	X_3 (KH_2PO_4)	X_4 ($MgSO_4$·$7H_2O$)	X_5 ($FeSO_4$·$7H_2O$)
−1	40 g/L	8 g/L	0.8 g/L	0.4 g/L	0.008 g/L
+1	60 g/L	12 g/L	1.2 g/L	0.6 g/L	0.0012 g/L

Table S2. The production and yield of emodin in the P-B design.

No.	variable					Emodin production (mg/L)	Emodin yield (mg/g)
	X_1	X_2	X_3	X_4	X_5		
1	1	1	−1	1	1	70.75 ± 2.52	4.19 ± 0.18
2	−1	1	1	−1	1	117.42 ± 1.37	8.20 ± 0.16
3	1	−1	1	1	−1	132.72 ± 1.56	6.55 ± 0.51
4	−1	1	−1	1	1	45.43 ± 1.15	4.15 ± 0.06
5	−1	−1	1	−1	1	94.70 ± 1.52	8.24 ± 0.19
6	−1	−1	−1	1	−1	83.19 ± 1.82	6.85 ± 0.26
7	1	−1	−1	−1	1	89.70 ± 3.98	4.72 ± 0.11
8	1	1	−1	−1	−1	88.05 ± 5.77	5.25 ± 0.38
9	1	1	1	−1	−1	128.59 ± 6.15	6.41 ± 0.23
10	−1	1	1	1	−1	113.52 ± 2.30	8.24 ± 0.07
11	1	−1	1	1	1	110.60 ± 4.42	5.78 ± 0.20
12	−1	−1	−1	−1	−1	122.49 ± 2.30	9.36 ± 0.74

Table S3. ANOVA for P-B design experiments of emodin production.

Factors	Sum of Squares	Mean Squares	*F*-Value	*p*-Value
Model	6058.75	1211.75	5.48	0.0306
X_1	158.76	158.76	0.72	0.4291
X_2	404.14	404.14	1.83	0.2250
X_3	3265.05	3265.05	14.78	0.0085
X_4	598.52	598.52	2.71	0.1509
X_5	1632.28	1632.28	7.39	0.0347

Table S4. ANOVA for P-B design experiments of emodin yield.

Factors	Sum of Squares	Mean Squares	*F*-Value	*p*-Value
Model	28.98	5.80	7.91	0.0128
X_1	12.29	12.29	16.78	0.0064
X_2	2.14	2.14	2.92	0.1385
X_3	6.58	6.58	8.98	0.0241
X_4	3.44	3.44	4.69	0.0735
X_5	4.53	4.53	6.18	0.0474

Table S5. The homology correlation of these genes between *A. favipes HN4-13* and the information from NCBI.

Gene	Accession	Identity
*emo*A	*Aspergillus terreus* NIH2624 alpha-1,2mannosyltransferase KTR1 (ATEG_09241) partial mRNA: XM_001217862.1	86.66%
	Aspergillus ibericus CBS 121593 glycosyl transferase (BO80DRAFT_163448), mRNA: XM_025713876.1	80.83%
*emo*B	*Aspergillus terreus* NIH2624 cytochrome c oxidase polypeptide VI, mitochondrial precursor (ATEG_09242) partial mRNA: XM_001217863.1	94.54%
	Aspergillus pseudonomius cytochrome c oxidase subunit VA-domain-containing protein (BDV37DRAFT_91687), mRNA: XM_032091441.1	90.76%
*emo*C		
*emo*D	*Aspergillus terreus* NIH2624 hypothetical protein (ATEG_09244) partial mRNA: XM_001217865.1	89.40%
	Aspergillus nomius NRRL 13137 elongation of fatty acids protein 1 (ANOM_010294), partial mRNA: XM_015555550.1	86.59%
*emo*E		
*emo*F	*Aspergillus terreus* NIH2624 conserved hypothetical protein (ATEG_08450) partial mRNA : XM_001217071.1	87.83%
	Aspergillus terreus NIH2624 conserved hypothetical protein (ATEG_08449) partial mRNA: XM_001217070.1	84.65%
*emo*G	*Aspergillus terreus* NIH2624 hypothetical protein (ATEG_08451) partial mRNA: XM_001217072.1	87.50%
	Aspergillus tanneri Type I Polyketide synthases (Type I PKS) (ATNIH1004_008023), partial mRNA: XM_033572636.1	75.02%
*emo*H	*Aspergillus terreus* NIH2624 hypothetical protein (ATEG_08454) partial mRNA: XM_001217592.1	89.32%
	Aspergillus terreus NIH2624 predicted protein (ATEG_08453) partial mRNA: XM_001217075.1	81.67%
*emo*I	*Paecilomyces divaricatus* MFS transporter (AgnL12), transcription factor (AgnL11), transcription factor (Agn10), transcriptional coactivator (AgnL9), dehydratase (AgnL8), hydrolase (AgnL7), reductase (AgnL6), hypothetical protein (AgnL5), oxidoreductase (AgnL4), Baeyer-Villiger monoxygenase (AgnL3), anthrone oxidase (AgnL2), decarboxylase (AgnL1), nr-PKS (Agnpks1), and oxidoreductase (AgnR1) genes, complete cds	91.67%
*emo*J	*Aspergillus terreus* NIH2624 predicted protein (ATEG_08456) partial mRNAemoJ-reverse: XM_001217594.1	86.10%
	Aspergillus fumigatus Af293 conserved hypothetical protein (AFUA_4G14460), partial mRNA: XM_746294.1	71.93%
*emo*K	*Aspergillus terreus* NIH2624 conserved hypothetical protein (ATEG_08457) partial mRNA: XM_001217595.1	92.67%
	Aspergillus terreus DHGO gene for dihydrogeodin oxidase, complete cds (exon1-7): D49538.1	88.76%
*emo*L	*Aspergillus terreus* NIH2624 conserved hypothetical protein (ATEG_08460) partial mRNA: XM_001217598.1	89.81%
	Aspergillus terreus NIH2624 predicted protein (ATEG_08459) partial mRNA: XM_001217597.1	87.81%

Table S6. Sequences of primer pairs for quantitative real-time RT-PCR (qRT-PCR) assay.

Target gene	Primer name	Primer sequence (5′-3′)
*emo*A	*emo*A-forword	TCATCTTGATCGCTCGGGTG
	*emo*A-reverse	ACGGAACCAGTGAACCTTGG
*emo*B	*emoB*-forword	AGTTCTCCGCCAGATTCGAG
	*emo*B-reverse	GAGGGGACAAGATCGTAGGC
*emo*C	*emo*C-forword	ATGGACCATACGGGAAGCTA
	*emo*C-reverse	AGGGGGTGTCTTGATTACTTGG
*emo*D	*emo*D-forword	ATGGCCCATCTTCGACAAGG
	*emo*D-reverse	GATGACTAGCATGGTGGCCG
*emo*E	*emo*E-forword	ATGTTGGGTAACACTCCCCG
	*emo*E-reverse	ACGATACCAGGTGTGGAAGC
*emo*F	*emo*F-forword	ACGGTTCCGGTCATCTTAGC
	*emo*F-reverse	GAACAGCTCATGGCTCGGTA
*emo*G	*emo*G-forword	GCGTCAACGTCTTGACCAAC
	*emo*G-reverse	TAACGAAACCACCCCGTCAG
*emo*H	*emo*H-forword	AGCCAATCCAAGTATGGGGC
	*emo*H-reverse	CCGTTTGGGTTGATTGCCAG
*emo*I	*emo*I-forword	GGCCCATTGACAGAGACGAC
	*emo*I-reverse	AGAAATCGCCGCTGTCCTAC
*emo*J	*emo*J-forword	TGTGGCAAGAGGTCGAAGAC
	*emo*J-reverse	TCAACCATCAACGTCCGTCC
*emo*K	*emo*K-forword	AGCCAGGTCGTGTTCAAGAG
	*emo*K-reverse	TCGTGTCGGCAAAGTTCTCA
*emo*L	*emo*L-forword	AACGCCAAACGAGAAGCCTA
	*emo*L-reverse	TTGGTCCCGTCGAAGATGTG
